# Author Correction: Regression-based Deep-Learning predicts molecular biomarkers from pathology slides

**DOI:** 10.1038/s41467-024-46298-5

**Published:** 2024-02-29

**Authors:** Omar S. M. El Nahhas, Chiara M. L. Loeffler, Zunamys I. Carrero, Marko van Treeck, Fiona R. Kolbinger, Katherine J. Hewitt, Hannah S. Muti, Mara Graziani, Qinghe Zeng, Julien Calderaro, Nadina Ortiz-Brüchle, Tanwei Yuan, Michael Hoffmeister, Hermann Brenner, Alexander Brobeil, Jorge S. Reis-Filho, Jakob Nikolas Kather

**Affiliations:** 1 Else Kroener Fresenius Center for Digital Health, Medical Faculty Carl Gustav Carus, TUD Dresden University of Technology, Dresden, Germany; 2 Department of Medicine 1, University Hospital and Faculty of Medicine Carl Gustav Carus, TUD Dresden University of Technology, Dresden, Germany; 3 Department of Visceral, Thoracic and Vascular Surgery, University Hospital and Faculty of Medicine Carl Gustav Carus, TUD Dresden University of Technology, Dresden, Germany; 4 University of Applied Sciences of Western Switzerland (HES-SO Valais), Rue du Technopole 3, 3960 Sierre, Valais Switzerland; 5 Centre d’Histologie, d’Imagerie et de Cytométrie (CHIC), Centre de Recherche des Cordeliers, INSERM, Sorbonne Université, Université Paris Cité, Paris, France; 6 Assistance Publique-Hôpitaux de Paris, Département de Pathologie, CHU Henri Mondor, F-94000 Créteil, France; 7 Institute of Pathology, University Hospital RWTH Aachen, Aachen, Germany; 8 Center for Integrated Oncology Aachen Bonn Cologne Duesseldorf (CIO ABCD), Cologne, Germany; 9 Division of Clinical Epidemiology and Aging Research, German Cancer Research Center (DKFZ), Heidelberg, Germany; 10 Division of Preventive Oncology, German Cancer Research Center (DKFZ) and National Center for Tumor Diseases (NCT), Heidelberg, Germany; 11 German Cancer Consortium (DKTK), German Cancer Research Center (DKFZ), Heidelberg, Germany; 12 Institute of Pathology, University Hospital Heidelberg, 69120 Heidelberg, Germany; 13 Tissue Bank, National Center for Tumor Diseases (NCT), University Hospital Heidelberg, 69120 Heidelberg, Germany; 14 Department of Pathology and Laboratory Medicine, Memorial Sloan Kettering Cancer Center, New York, NY USA; 15 Pathology & Data Analytics, Leeds Institute of Medical Research at St James’s, University of Leeds, Leeds, United Kingdom; 16 Medical Oncology, National Center for Tumor Diseases (NCT), University Hospital Heidelberg, Heidelberg, Germany

**Keywords:** Image processing, Prognostic markers, Machine learning, Cancer imaging, Tumour biomarkers

Correction to: *Nature Communications* 10.1038/s41467-024-45589-1, published online 10 February 2024

In this article the affiliation details for Mara Graziani were incorrectly given as ‘University of Applied Sciences of Western Switzerland (HES-SO Valais), Rue du Technopole 3, 3960, Sierre, Valais, Switzerland’ and ‘IBM Research Europe, 8803, Rüschlikon, Switzerland’, but should have been ‘University of Applied Sciences of Western Switzerland (HES-SO Valais), Rue du Technopole 3, 3960, Sierre, Valais, Switzerland’.

The original article has been corrected.

