# Supplementary material for: Regression-based Deep-Learning predicts molecular biomarkers from pathology slides
Source: Nat Commun. 2024 Feb 10;15:1253. doi: 10.1038/s41467-024-45589-1 (PMC10858881; doi:10.1038/s41467-024-45589-1)
Supplement: Supplementary file 1 — Supplementary Information [file 41467_2024_45589_MOESM1_ESM.pdf]

Supplements

Cohort	CAMIL regression<br>AUROC 95%CI	Graziani et al. regression<br>AUROC 95%CI	CAMIL classification<br>AUROC 95%CI
TCGA-BRCA	0.78 [0.75 - 0.81]	0.7 [0.66 - 0.73]	0.74 [0.71 - 0.78]
TCGA-CRC	0.76 [0.65 - 0.87]	0.47 [0.36 - 0.57]	0.73 [0.6 - 0.85]
TCGA-GBM	0.64 [0.37 - 0.79]	0.64 [0.41 - 0.74]	0.52 [0.28 - 0.76]
TCGA-LUAD	0.72 [0.67 - 0.77]	0.71 [0.66 - 0.76]	0.7 [0.65 - 0.75]
TCGA-LUSC	0.57 [0.52 - 0.63]	0.57 [0.51 - 0.62]	0.57 [0.52 - 0.63]
TCGA-PAAD	0.72 [0.62 - 0.81]	0.4 [0.26 - 0.53]	0.54 [0.43 - 0.65]
TCGA-UCEC	0.82 [0.78 - 0.86]	0.67 [0.6 - 0.75]	0.73 [0.67 - 0.8]
CPTAC-LUAD	0.81 [0.77 - 0.85]	0.83 [0.79 - 0.87]	0.82 [0.78 - 0.86]
CPTAC-LUSC	0.62 [0.56 - 0.67]	0.66 [0.61 - 0.7]	0.62 [0.57 - 0.67]
CPTAC-PAAD	0.68 [0.56 - 0.79]	0.55 [0.47 - 0.63]	0.6 [0.49 - 0.71]
CPTAC-UCEC	0.96 [0.93 - 0.98]	0.89 [0.79 - 0.98]	0.98 [0.96 - 0.99]

Suppl. Table 1: Area under the receiver operating characteristics (AUROC) with 95% confidence interval (CI) and corresponding p-values of the homologous recombination deficiency (HRD) models trained with site-aware splits. The evaluation AUROC and AUPRC for this CAMIL classification, Graziani et al. regression and CAMIL regression is calculated for the median ensemble of each model trained on the HRD score. In The Cancer Genome Atlas (TCGA), breast cancer (BRCA), colorectal cancer (CRC), glioblastoma (GBM), lung adenocarcinoma (LUAD), lung squamous cell cancer (LUSC), pancreatic adenocarcinoma (PAAD) and endometrial cancer (UCEC) were used for site-aware training. In the Clinical Proteomic Tumor Analysis Consortium (CPTAC) effort, LUAD, LUSC, PAAD and UCEC were used as external validation cohorts. Statistically insignificant AUROCs have a 95% CI crossing 0.5.

Cohort	CAMIL regression vs Graziani et al. regression<br>p-value	CAMIL regression vs CAMIL classification<br>p-value	CAMIL classification vs Graziani et al. regression<br>p-value
TCGA-BRCA	0.01	0.02	0.07
TCGA-CRC	0.06	0.75	0.01
TCGA-GBM	NA	NA	NA
TCGA-LUAD	0.30	0.17	0.98
TCGA-LUSC	0.73	0.55	0.94
TCGA-PAAD	0.90	0.31	0.64
TCGA-UCEC	0.04	0.42	0.32
CPTAC-LUAD	0.33	0.55	0.84
CPTAC-LUSC	0.66	0.98	0.71
CPTAC-PAAD	0.98	0.06	0.52
CPTAC-UCEC	0.37	0.44	0.03

Suppl. Table 2: Results of the DeLong's test for comparison of the Area Under the Receiver Operating Characteristics (AUROC) of the homologous recombination deficiency (HRD) models trained with site-aware splits. In The Cancer Genome Atlas (TCGA), breast cancer (BRCA), colorectal cancer (CRC), glioblastoma (GBM), lung adenocarcinoma (LUAD), lung squamous cell cancer (LUSC), pancreatic adenocarcinoma (PAAD) and endometrial cancer (UCEC) were used for site-aware training. In the Clinical Proteomic Tumor Analysis Consortium (CPTAC) effort, LUAD, LUSC, PAAD and UCEC were used as external validation cohorts. The performed statistical test is the two-sided DeLong's test, with Bonferroni corrections for multiple testing. A p-value ≤ 0.0167 indicates a significant difference in the AUROCs. The test for TCGA-GBM could not be performed due to only 1 HRD+ case being present in the dataset.

	CAMIL classification				CAMIL regression					
	HRD+ median	HRD+ IQR	HRD- median	HRD- IQR	HRD+ median	HRD+ IQR	HRD- median	HRD- IQR	Absolute improvement using CAMIL regression	Mean absolute improvement using CAMIL regression
TCGA-BRCA	0.63	0.37	0.41	0.33	0.60	0.41	0.27	0.36	11.2%	
TCGA-CRC	0.45	0.11	0.43	0.08	0.70	0.13	0.51	0.33	17.4%	
TCGA-GBM	0.79	0.23	0.80	0.29	0.32	0.30	0.49	0.24	16.3%	
TCGA-LUAD	0.58	0.24	0.45	0.27	0.61	0.25	0.43	0.32	4.9%	
TCGA-LUSC	0.52	0.21	0.50	0.19	0.54	0.31	0.49	0.30	3.0%	
TCGA-PAAD	0.60	0.34	0.57	0.36	0.71	0.13	0.59	0.24	8.2%	
TCGA-UCEC	0.65	0.27	0.47	0.29	0.45	0.37	0.18	0.28	8.7%	9.9%
CPTAC-LUAD	0.67	0.21	0.49	0.33	0.65	0.16	0.47	0.27	-0.1%	
CPTAC-LUSC	0.55	0.19	0.52	0.23	0.54	0.25	0.47	0.26	5.1%	
CPTAC-PAAD	0.78	0.41	0.55	0.45	0.50	0.30	0.36	0.26	-8.7%	
CPTAC-UCEC	0.77	0.21	0.45	0.33	0.70	0.11	0.15	0.23	23.2%	4.9%

	Graziani et al. regression				CAMIL regression					
	HRD+ median	HRD+ IQR	HRD- median	HRD- IQR	HRD+ median	HRD+ IQR	HRD- median	HRD- IQR	Absolute improvement using CAMIL regression	Mean absolute improvement using CAMIL regression
TCGA-BRCA	0.51	0.33	0.33	0.27	0.60	0.41	0.27	0.36	15.3%	
TCGA-CRC	0.52	0.27	0.52	0.25	0.70	0.13	0.51	0.33	18.9%	
TCGA-GBM	0.73	0.30	0.55	0.31	0.32	0.30	0.49	0.24	-0.3%	
TCGA-LUAD	0.57	0.27	0.46	0.30	0.61	0.25	0.43	0.32	6.3%	
TCGA-LUSC	0.55	0.46	0.55	0.48	0.54	0.31	0.49	0.30	4.6%	
TCGA-PAAD	0.49	0.71	0.71	0.56	0.71	0.13	0.59	0.24	-10.9%	
TCGA-UCEC	0.49	0.56	0.34	0.34	0.45	0.37	0.18	0.28	12.3%	6.6%
CPTAC-LUAD	0.78	0.20	0.57	0.32	0.65	0.16	0.47	0.27	-2.4%	
CPTAC-LUSC	0.32	0.66	0.32	0.67	0.54	0.25	0.47	0.26	7.7%	
CPTAC-PAAD	0.75	0.61	0.68	0.62	0.50	0.30	0.36	0.26	6.7%	
CPTAC-UCEC	0.74	0.41	0.45	0.51	0.70	0.11	0.15	0.23	26.2%	9.5%

Suppl. Table 3: Median and interquartile range (IQR) for the CAMIL classification, CAMIL regression and Graziani et al. regression approach. The median and IQR is calculated for each model trained on the homologous recombination deficiency (HRD) score. Normalization is performed to ensure a consistent scale for comparison across the different methods' prediction scores. The predicted scores are min-max normalized with 95% of the data falling in between the 2.5th and 97.5th percentile, removing extreme values that potentially distort the scaling. A positive percentage indicates a larger distance between the median peaks of the groups' distribution using CAMIL regression, whereas a negative percentage indicates a larger distance between the median peaks of the groups' distribution using the compared approach. In The Cancer Genome Atlas (TCGA), breast cancer (BRCA), colorectal cancer (CRC), glioblastoma (GBM), lung adenocarcinoma (LUAD), lung squamous cell cancer (LUSC), pancreatic adenocarcinoma (PAAD) and endometrial cancer (UCEC) were used for site-aware training. In the Clinical Proteomic Tumor Analysis Consortium (CPTAC) effort, LUAD, LUSC, PAAD and UCEC were used as external validation cohorts.

	Graziani et al. regression		CAMIL regression		Both methods
	Pearson’s r	p-value	Pearson’s r	p-value	n samples
TCGA-BRCA	0.359515	p=6.21E-22	0.530024	p=6.05E-50	672
TCGA-CRC	0.044258	p=0.41	0.223784	p=2.88E-05	343
TCGA-GBM	0.090259	p=0.34	0.242396	p=8.75E-03	116
TCGA-LUAD	0.324392	p=9.28E-07	0.439135	p=9.72E-12	219
TCGA-LUSC	0.079944	p=0.20	0.155889	p=0.01	255
TCGA-PAAD	-0.05968	p=0.55	0.269659	p=6.39E-03	101
TCGA-UCEC	0.28976	p=7.39E-07	0.554872	p=3.59E-24	282
CPTAC-LUAD	0.494131	p=7.33E-08	0.566941	p=2.36E-10	106
CPTAC-LUSC	0.323326	p=6.42E-04	0.18614	p=0.05	108
CPTAC-PAAD	0.080853	p=0.34	0.057652	p=0.50	139
CPTAC-UCEC	0.509284	p=7.32E-08	0.785403	6.35E-22	99

Suppl. Table 4: Comparison of Graziani regression with CAMIL regression through the Pearson’s r. Pearson’s r ranges between -1 and 1, where a correlation closer to an extreme indicates a better regression model. The performed statistical test is the two-sided t-test. Significance is reached at $p \leq 0.05$, indicating the correlation is nonzero following the Scipy implementation. In The Cancer Genome Atlas (TCGA), breast cancer (BRCA), colorectal cancer (CRC), glioblastoma (GBM), lung adenocarcinoma (LUAD), lung squamous cell cancer (LUSC), pancreatic adenocarcinoma (PAAD) and endometrial cancer (UCEC) were used for site-aware training. In the Clinical Proteomic Tumor Analysis Consortium (CPTAC) effort, LUAD, LUSC, PAAD and UCEC were used as external validation cohorts. The models are trained on the homologous recombination deficiency (HRD) score, for which the median prediction scores of the 5-fold models were taken for the calculation of the metrics. Therefore, the metrics were calculated with a single prediction for each patient.

Supplementary Note 1: CAMIL regression has better generalization capabilities than Graziani regression

Given the nature of how AUROCs are produced, the continuous output score of the regression models can be used in combination with a categorical target, such as the clinically-relevant binarized HRD score. However, AUROCs only indicate how well the given continuous score is able to separate between the negative and positive class, i.e. rewarding a high AUROC for a model which outputs a low intra-class variance and a high inter-class variance, regardless of the absolute range of the prediction scores. Analyzing the performance between regression methods, it was found that CAMIL regression is capable of predicting more clinically-relevant output scores which are closer to the absolute ground-truth (**Suppl. Fig. 1)**, giving a prediction range of (30, 34) and (10, 50) for the Graziani et al. regression model and our CAMIL regression model on the CPTAC-LUAD external test cohort, respectively. For this analysis, LUAD was chosen as it resulted in the only statistically significant tumor-type for the regression models with both an internal and external validation set for the HRD target, while having similar performance metrics (**Suppl. Table 1 and 4**). With a Pearson's r of 0.49 ($p \le 0.0001$) for the Graziani et al. regression and a Pearson's r of 0.57 ($p \le 0.0001$) for our proposed CAMIL regression model, superior generalization capabilities for our proposed CAMIL regression over the regression method by Graziani et al. are observed. However, the measure by AUROC would indicate that the regression by Graziani et al. has superior performance, showing the limited capabilities of comparing regression models solely through AUROC values.

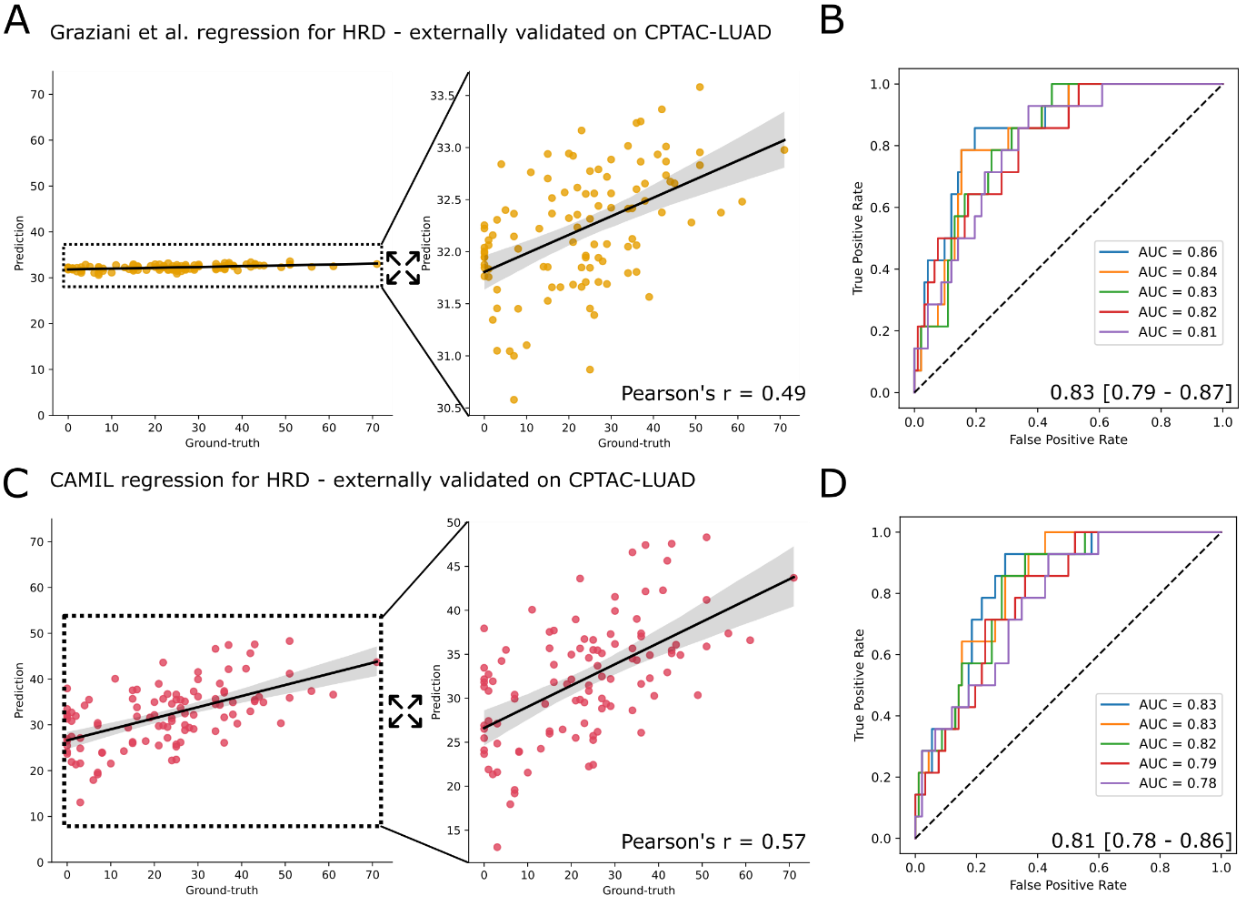

Suppl. Fig. 1: Comparison of CAMIL regression and Graziani et al. regression on an external testing cohort. A) The correlation plot of the regression approach by Graziani et al. on the external cohort of lung adenocarcinoma (LUAD) from the Clinical Proteomic Tumor Analysis Consortium (CPTAC) effort in the original range of the homologous recombination deficiency (HRD) continuous ground-truth, and a zoom-in of the same data in the range of the model's prediction. B) The

corresponding area under the receiver operator characteristic (AUROC) curve using the continuous prediction scores plotted in panel A and the HRD binary ground-truth. C) The correlation plot of the CAMIL regression approach from this study on the external cohort CPTAC-LUAD in the original range of the HRD continuous ground-truth, and a zoom-in of the same data in the range of the model's prediction. D) The corresponding AUROC curve using the continuous prediction scores plotted in panel C and the HRD binary ground-truth. The yellow color indicates predictions made by the model trained using the method by Graziani et al., whereas the red color indicates predictions made by the CAMIL regression model. The sample size to calculate the metrics is n=106 independent patient samples. Source data are provided as a Source Data file.

	CAMIL classification	Graziani et al. regression	CAMIL regression
batch size	64	1	1
batch normalization	Yes	No	No
optimizer	Adam	Stochastic gradient descent	Adam
loss function	Weighted Cross-entropy	Mean Squared Error	Weighted Mean Squared Error
epochs	25	100	25
dropout	50%	20%	0%
target balancing	Inverse weighted	No	Kernel-based[54]
Base model	ImageNet weighted ResNet50 + contrastive clustering[33]	ImageNet weighted ResNet18	ImageNet weighted ResNet50 + contrastive clustering[33]

Suppl. Table 5: Overview of the differences between the three modeling approaches. The main differences between contrastively-clustered attention-based multiple instance learning (CAMIL) classification, Graziani et al. regression and CAMIL regression.

For deeper analysis into the differences between the regression heads of Graziani et al. regression and CAMIL regression, an ablation study (**Suppl. Table 7)** was performed on CAMIL regression using the TCGA-BRCA cohort. The TCGA-BRCA cohort was chosen for the ablation study as both Graziani et al. regression model and the CAMIL regression model gave statistically significant AUROCs for all 5 folds which were in a similar range with low variance, in contrast to TCGA-LUAD which showed more variance among the 5 folds due to an outlying fold (**Fig. 2**). Similarly to previous experiments, the median prediction score across the 5 folds was used for statistical analyses.

	[0,91] prediction range	[0,1] Pearson's r	[0,1] AUROC 95% CI	n samples
CAMIL regression	(6.68, 69.71)	0.53 (p≤0.0001)	0.78 [0.75 - 0.81]	672
With 20% dropout	(7.03, 70.09)	0.53 (p≤0.0001)	0.78 [0.74 - 0.81]	672
With SGD	(33.71, 37.12)	0.07 (p=0.07)	0.62 [0.58 - 0.66]	672
With 100 epochs	(6.08, 67.89)	0.54 (p≤0.0001)	0.78 [0.75 - 0.81]	672
Without kernel-based balancing	(6.91, 68.93)	0.52 (p≤0.0001)	0.78 [0.75 - 0.81]	672

Suppl. Table 6: Ablation study of CAMIL regression with adaptations from Graziani et al. regression for the test set of The Cancer Genome Atlas (TCGA) breast cancer cohort for homologous recombination deficiency (HRD). Using the same site-aware splits for all the models, our CAMIL regression modeling approach was altered according to the changes found in Graziani et al. regression approach, adding a layer with 20% dropout, swapping the Adam optimizer for stochastic gradient descent (SGD), increasing the epochs to 100, and removing the kernel-based balancing. The

regression approaches are compared through their prediction score range between [0, 91], where a wider range is better, the Pearson's r, and the area under the receiver operating characteristic (AUROC) with 95% confidence interval (CI) across all five folds. The models are trained on the HRD score, for which the median prediction scores of the 5-fold models were taken for the calculation of the metrics. Therefore, the metrics were calculated with a single prediction for each patient.

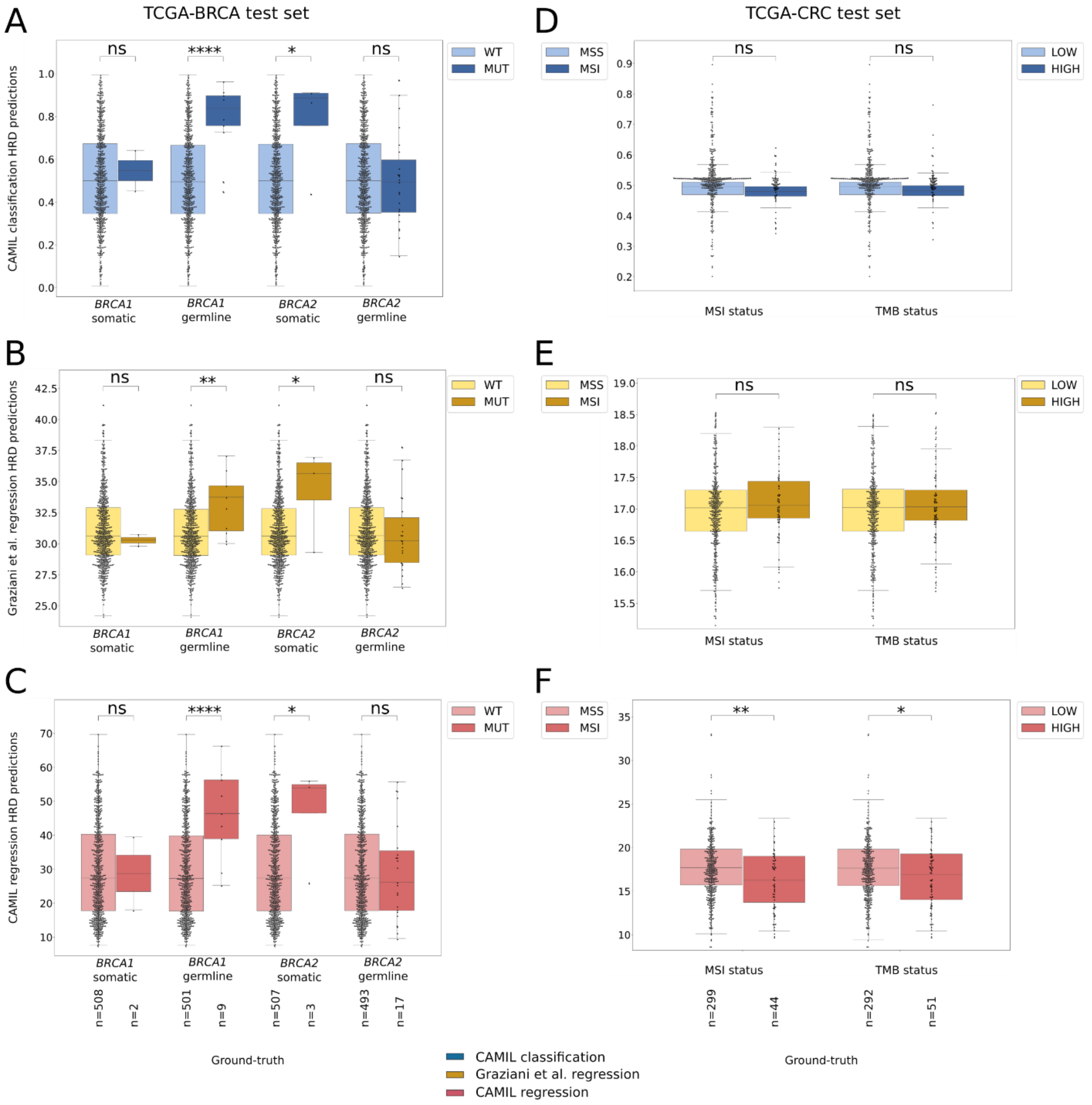

Suppl. Fig. 2: Concordance analysis of homologous recombination deficiency (HRD) predicted scores from the three approaches. In The Cancer Genome Atlas (TCGA), the breast cancer (BRCA) and colorectal cancer (CRC) cohorts were used for site-aware training for the three approaches. Concordance with the predicted HRD score was analyzed with BRCA1 and BRCA2 somatic/germline mutations in TCGA-BRCA in panel A) to C), and microsatellite instability (MSI) status and tumor mutational burden (TMB) status in TCGA-CRC in panel D) to F). An independent two-sided t-test was performed to measure if the means of the groups per category were significantly different. Significance is indicated by * p≤0.05, ** p≤0.01, *** p≤0.001, **** p≤0.0001, or alternatively by ns for p>0.05. The box plot represents the interquartile range (IQR), with the lower, middle and upper edge being the 25th, 50th, and 75th percentile. The whiskers of the box plots are defined as the minimum and maximum values 1.5 times the IQR away from the lower and upper quartiles of the data, respectively. The pathogenic germline mutation data for TCGA-BRCA were provided by Memorial Sloan Kettering and is limited to the intersection of cases which have all variables available to perform the concordance analysis, and are indicated with the number (n) of cases. The n cases are consistent for every modeling approach. Source data are provided as a Source Data file.

CAMIL regression AUROC

	TCGA-BRCA	TCGA-CRC	TCGA-LIHC	TCGA-LUAD	TCGA-LUSC	TCGA-STAD	TCGA-UCEC
**TIL Regional Fraction**	0.88 [0.86 - 0.91]	0.79 [0.75 - 0.84]	NA	0.87 [0.83 - 0.9]	0.88 [0.84 - 0.93]	0.79 [0.73 - 0.85]	0.82 [0.78 - 0.86]
**Proliferation**	0.84 [0.81 - 0.86]	0.59 [0.51 - 0.66]	0.87 [0.82 - 0.91]	0.84 [0.79 - 0.88]	0.78 [0.71 - 0.85]	0.87 [0.82 - 0.92]	0.73 [0.68 - 0.79]
**Leukocyte Fraction**	0.8 [0.77 - 0.83]	0.76 [0.72 - 0.81]	0.8 [0.75 - 0.84]	0.73 [0.68 - 0.78]	0.72 [0.66 - 0.78]	0.72 [0.67 - 0.78]	0.77 [0.73 - 0.81]
**LISS**	0.8 [0.78 - 0.83]	0.7 [0.66 - 0.74]	0.7 [0.64 - 0.76]	0.65 [0.58 - 0.72]	0.74 [0.69 - 0.79]	0.67 [0.61 - 0.72]	0.73 [0.69 - 0.77]
**Stromal Fraction**	0.81 [0.78 - 0.83]	0.68 [0.63 - 0.73]	0.77 [0.72 - 0.83]	0.76 [0.7 - 0.83]	0.77 [0.72 - 0.82]	0.62 [0.55 - 0.68]	0.67 [0.61 - 0.72]

CAMIL classification AUROC

	TCGA-BRCA	TCGA-CRC	TCGA-LIHC	TCGA-LUAD	TCGA-LUSC	TCGA-STAD	TCGA-UCEC
TIL Regional Fraction	0.82 [0.79 - 0.85]	0.82 [0.79 - 0.85]	NA	0.83 [0.79 - 0.87]	0.84 [0.79 - 0.89]	0.8 [0.74 - 0.86]	0.78 [0.74 - 0.83]
Proliferation	0.82 [0.8 - 0.85]	0.58 [0.49 - 0.66]	0.78 [0.71 - 0.84]	0.8 [0.75 - 0.85]	0.78 [0.71 - 0.85]	0.7 [0.62 - 0.77]	0.69 [0.63 - 0.75]
Leukocyte Fraction	0.73 [0.7 - 0.76]	0.74 [0.69 - 0.79]	0.79 [0.75 - 0.84]	0.71 [0.65 - 0.77]	0.73 [0.67 - 0.78]	0.73 [0.68 - 0.79]	0.73 [0.69 - 0.78]
LISS	0.73 [0.7 - 0.77]	0.68 [0.64 - 0.73]	0.63 [0.57 - 0.7]	0.61 [0.53 - 0.68]	0.69 [0.64 - 0.74]	0.66 [0.6 - 0.71]	0.67 [0.62 - 0.71]
Stromal Fraction	0.76 [0.73 - 0.79]	0.61 [0.56 - 0.67]	0.73 [0.67 - 0.79]	0.75 [0.69 - 0.81]	0.76 [0.71 - 0.81]	0.61 [0.54 - 0.67]	0.61 [0.56 - 0.67]

Graziani et al. regression AUROC

	TCGA-BRCA	TCGA-CRC	TCGA-LIHC	TCGA-LUAD	TCGA-LUSC	TCGA-STAD	TCGA-UCEC
TIL Regional Fraction	0.69 [0.65 - 0.72]	0.62 [0.56 - 0.68]	NA	0.76 [0.71 - 0.8]	0.67 [0.61 - 0.74]	0.7 [0.62 - 0.77]	0.68 [0.63 - 0.73]
Proliferation	0.76 [0.73 - 0.79]	0.55 [0.44 - 0.66]	0.64 [0.57 - 0.7]	0.76 [0.71 - 0.8]	0.68 [0.61 - 0.75]	0.66 [0.6 - 0.71]	0.59 [0.53 - 0.65]
Leukocyte Fraction	0.66 [0.62 - 0.69]	0.65 [0.57 - 0.73]	0.68 [0.63 - 0.74]	0.68 [0.62 - 0.74]	0.59 [0.53 - 0.65]	0.66 [0.61 - 0.72]	0.52 [0.47 - 0.57]
LISS	0.69 [0.66 - 0.73]	0.55 [0.49 - 0.62]	0.55 [0.49 - 0.62]	0.61 [0.53 - 0.68]	0.58 [0.52 - 0.64]	0.57 [0.51 - 0.63]	0.53 [0.48 - 0.58]
Stromal Fraction	0.68 [0.65 - 0.72]	0.59 [0.51 - 0.67]	0.73 [0.67 - 0.79]	0.68 [0.61 - 0.75]	0.71 [0.65 - 0.76]	0.62 [0.55 - 0.68]	0.5 [0.44 - 0.56]

Suppl. Table 7: Area under the receiver operating curve (AUROC) with 95% confidence interval (CI) of biological process models from site-aware training. The performance of CAMIL classification and CAMIL regression models with site-aware splits is measured on cohorts from The Cancer Genome Atlas (TCGA), breast cancer (BRCA), colorectal cancer (CRC), liver hepatocellular carcinoma (LIHC), lung adenocarcinoma (LUAD), lung squamous cell cancer (LUSC), gastric cancer (STAD) and endometrial cancer (UCEC) on biomarkers for tumor infiltrating lymphocytes (TIL) regional fraction, proliferation, leukocyte fraction, lymphocyte infiltration signature score, and stromal fraction. The performance metric is the AUROC with corresponding 95%CI. Statistically insignificant AUROCs have a 95% CI crossing 0.5. No data for analysis was available for the TIL regional fraction biomarker in TCGA-LIHC.

Delta AUROC CAMIL regression – CAMIL classification

	TCGA-BRCA	TCGA-CRC	TCGA-LIHC	TCGA-LUAD	TCGA-LUSC	TCGA-STAD	TCGA-UCEC
TIL Regional Fraction	0.06	-0.03	NA	0.04	0.04	-0.01	0.04
Proliferation	0.04	0.01	0.09	0.04	0.00	0.17	0.04
Leukocyte Fraction	0.10	0.02	0.01	0.02	-0.01	-0.03	0.04
LISS	0.10	0.02	0.10	0.04	0.05	0.07	0.06
Stromal Fraction	0.05	0.07	0.04	0.01	0.01	0.01	0.06

Delta AUROC CAMIL regression – Graziani et al. regression

	TCGA-BRCA	TCGA-CRC	TCGA-LIHC	TCGA-LUAD	TCGA-LUSC	TCGA-STAD	TCGA-UCEC
TIL Regional Fraction	0.19	0.17	NA	0.11	0.21	0.09	0.14
Proliferation	0.08	0.04	0.23	0.08	0.10	0.21	0.14
Leukocyte Fraction	0.14	0.11	0.12	0.05	0.13	0.04	0.25
LISS	0.11	0.15	0.15	0.04	0.16	0.10	0.20
Stromal Fraction	0.13	0.09	0.04	0.08	0.06	0.00	0.17

Delta AUROC CAMIL classification – Graziani et al. regression

	TCGA-BRCA	TCGA-CRC	TCGA-LIHC	TCGA-LUAD	TCGA-LUSC	TCGA-STAD	TCGA-UCEC
TIL Regional Fraction	0.13	0.20	NA	0.07	0.17	0.10	0.10
Proliferation	0.04	0.03	0.14	0.04	0.10	0.04	0.10
Leukocyte Fraction	0.04	0.09	0.11	0.03	0.14	0.07	0.21
LISS	0.01	0.13	0.05	0.00	0.11	0.03	0.14
Stromal Fraction	0.08	0.02	0.00	0.07	0.05	-0.01	0.11

Suppl. Table 8: Deltas of the Area under the receiver operating curve (AUROC) of biological process models from site-aware training. The performance of CAMIL classification, Graziani et al. regression and CAMIL regression models with site-aware splits is measured on cohorts from The Cancer Genome Atlas (TCGA), breast cancer (BRCA), colorectal cancer (CRC), liver hepatocellular carcinoma (LIHC), lung adenocarcinoma (LUAD), lung squamous cell cancer (LUSC), gastric cancer (STAD) and endometrial cancer (UCEC) on biomarkers for tumor infiltrating lymphocytes (TIL) regional fraction, proliferation, leukocyte fraction, lymphocyte infiltration signature score, and stromal fraction. The performance metric is the AUROC. Statistically insignificant deltas are measured by a paired two-tailed DeLong's test. No data for analysis was available for the TIL regional fraction biomarker in TCGA-LIHC.

Paired two-tailed DeLong’s test CAMIL regression – CAMIL classification

	TCGA-BRCA	TCGA-CRC	TCGA-LIHC	TCGA-LUAD	TCGA-LUSC	TCGA-STAD	TCGA-UCEC
TIL Regional Fraction	1.19E-02	7.84E-01	NA	6.41E-01	1.73E-01	2.56E-01	9.20E-02
Proliferation	7.63E-01	7.58E-03	1.35E-02	2.30E-01	2.31E-01	1.81E-01	1.87E-01
Leukocyte Fraction	1.75E-02	4.70E-01	4.67E-01	3.87E-01	7.02E-01	2.75E-01	2.12E-01
LISS	1.24E-02	8.21E-02	3.75E-01	5.77E-02	1.19E-01	1.07E-01	4.37E-01
Stromal Fraction	1.05E-01	4.56E-01	8.70E-01	2.40E-01	2.41E-01	9.16E-01	7.98E-02

Paired two-tailed DeLong’s test CAMIL regression – Graziani et al. regression

	TCGA-BRCA	TCGA-CRC	TCGA-LIHC	TCGA-LUAD	TCGA-LUSC	TCGA-STAD	TCGA-UCEC
TIL Regional Fraction	9.07E-06	5.86E-03	NA	1.20E-03	1.68E-03	3.43E-02	3.74E-04
Proliferation	2.35E-01	4.21E-01	2.96E-03	7.97E-03	3.82E-01	7.59E-02	8.40E-03
Leukocyte Fraction	3.69E-07	2.33E-01	1.51E-02	1.98E-01	1.52E-01	9.26E-01	1.63E-01
LISS	1.09E-02	2.66E-01	6.17E-01	3.29E-02	8.84E-03	7.94E-01	5.41E-03
Stromal Fraction	1.00E-03	4.53E-02	6.26E-01	8.70E-01	3.38E-01	5.44E-01	3.53E-01

Paired two-tailed DeLong’s test CAMIL classification – Graziani et al. regression

	TCGA-BRCA	TCGA-CRC	TCGA-LIHC	TCGA-LUAD	TCGA-LUSC	TCGA-STAD	TCGA-UCEC
TIL Regional Fraction	2.71E-03	1.73E-03	NA	1.47E-02	3.37E-02	1.73E-01	2.37E-02
Proliferation	2.90E-01	5.70E-01	1.38E-01	1.68E-01	7.82E-02	2.20E-01	1.39E-01
Leukocyte Fraction	1.41E-04	1.60E-01	6.93E-02	6.17E-02	2.22E-01	2.51E-01	5.69E-01
LISS	5.56E-01	9.41E-01	2.29E-01	1.58E-01	8.19E-02	1.38E-01	3.89E-02
Stromal Fraction	1.35E-02	3.40E-01	6.72E-01	5.92E-01	9.34E-01	7.28E-01	7.79E-01

Suppl. Table 9: Results of the DeLong’s test for comparison of the Area Under the Receiver Operating Characteristics (AUROC) of biological process models trained from site-aware training. The performance of CAMIL classification, Graziani et al. regression and CAMIL regression models with site-aware splits is measured on cohorts from The Cancer Genome Atlas (TCGA), breast cancer (BRCA), colorectal cancer (CRC), liver hepatocellular carcinoma (LIHC), lung adenocarcinoma (LUAD), lung squamous cell cancer (LUSC), gastric cancer (STAD) and endometrial cancer (UCEC) on biomarkers for tumor infiltrating lymphocytes (TIL) regional fraction, proliferation, leukocyte fraction, lymphocyte infiltration signature score, and stromal fraction. Statistically insignificant deltas are measured by a paired two-tailed DeLong’s test. A p-value ≤ 0.0167 indicates a significant difference in the AUROCs No data for analysis was available for the TIL regional fraction biomarker in TCGA-LIHC.

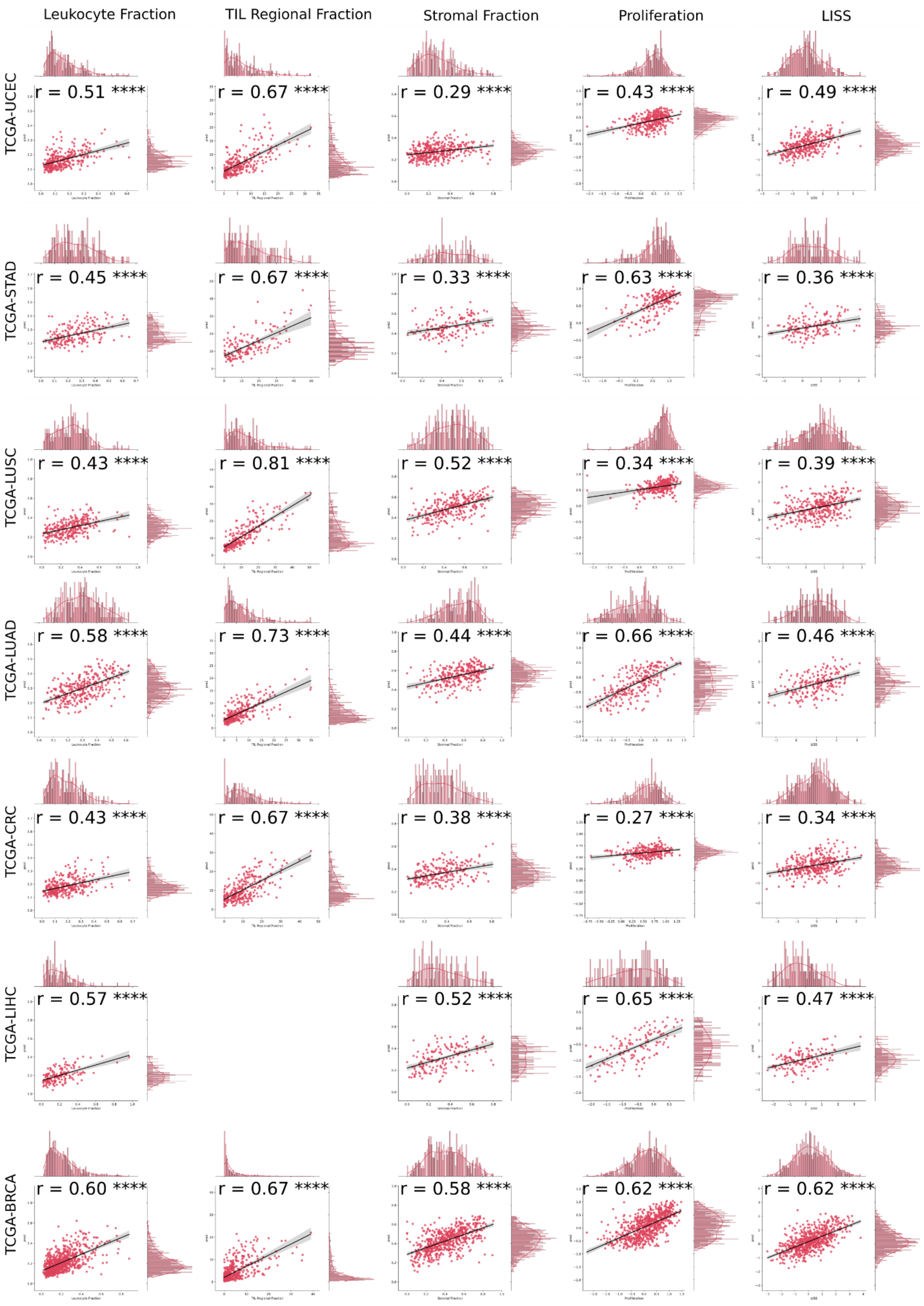

Suppl. Fig. 3: Distribution and correlation plots of the biological process models from CAMIL regression. The performance of CAMIL regression models with site-aware splits is measured on

cohorts from The Cancer Genome Atlas (TCGA), breast cancer (BRCA), colorectal cancer (CRC), liver hepatocellular carcinoma (LIHC), lung adenocarcinoma (LUAD), lung squamous cell cancer (LUSC), gastric cancer (STAD) and endometrial cancer (UCEC) on biomarkers for tumor infiltrating lymphocytes (TIL) regional fraction, proliferation, leukocyte fraction, lymphocyte infiltration signature score (LISS), and stromal fraction. Pearson's r ranges between -1 and 1, where a correlation closer to an extreme indicates a better regression model. The performed statistical test is the two-sided t-test. Significance is reached at $p \leq 0.05$, indicating the correlation is nonzero following the Scipy implementation. Significance is indicated by * $p \leq 0.05$, ** $p \leq 0.01$, *** $p \leq 0.001$, **** $p \leq 0.0001$, or alternatively by ns for $p > 0.05$. Source data are provided as a Source Data file. The red color indicates predictions made by the CAMIL regression model. The analysis is performed with independent patient samples, with the number of independent samples indicated within brackets for proliferation, BRCA (687), CRC (266), LIHC (131), LUAD (251), LUSC (280), STAD (201), UCEC (345), for LISS, BRCA (630), CRC (356), LIHC (153), LUAD (201), LUSC (340), STAD (158), UCEC (324), for TIL regional fraction, BRCA (655), CRC (276), LUSC (223), LUAD (269), STAD (149), UCEC (272), for stromal fraction, BRCA (589), CRC (234), LIHC (157), LUAD (294), LUSC (287), STAD (152), UCEC (384), and for leukocyte fraction, BRCA (763), CRC (312), LIHC (159), LUAD (323), LUSC (289), STAD (193), UCEC (317).

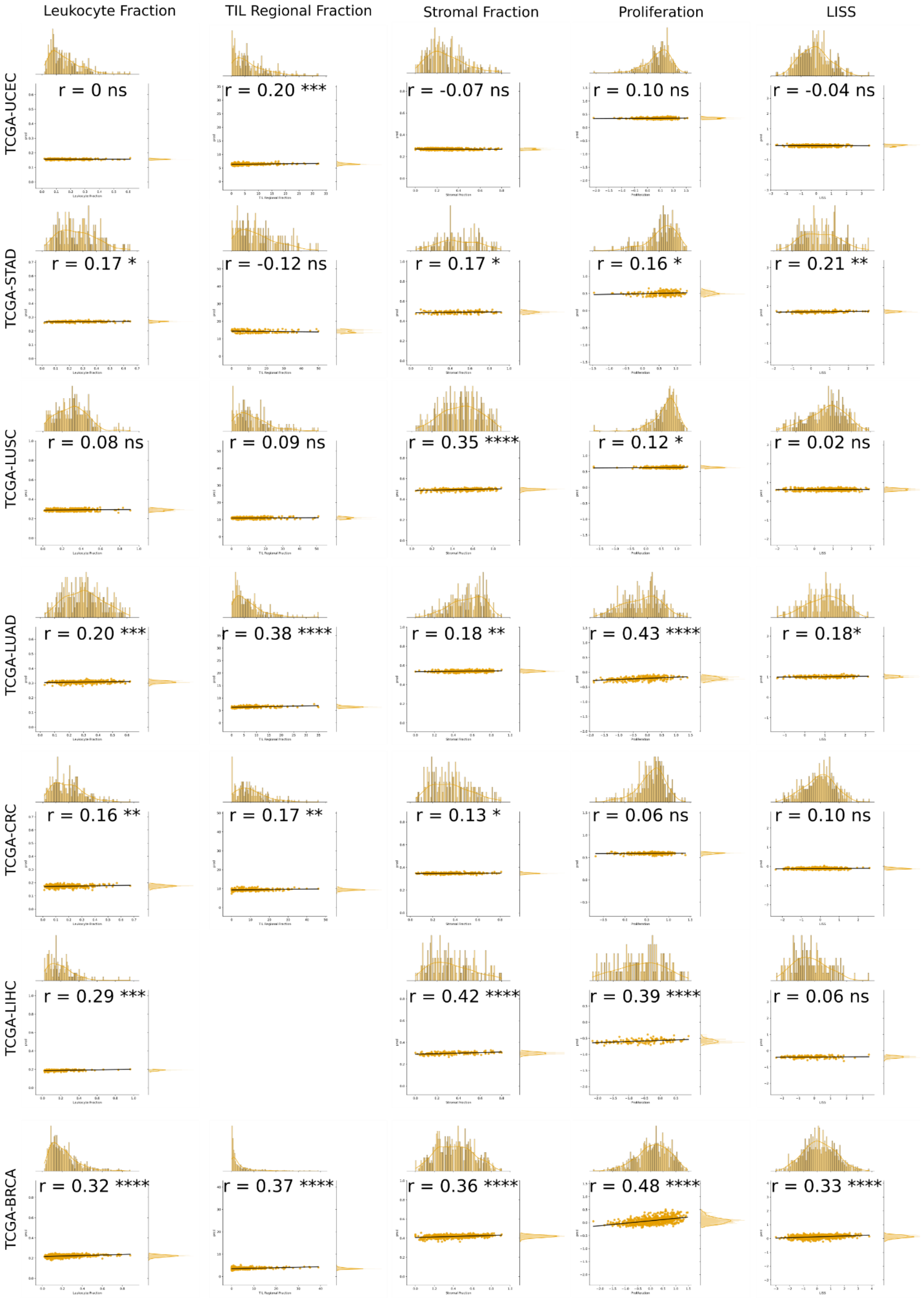

Suppl. Fig. 4: Distribution and correlation plots of the biological process models from Graziani et al. regression. The performance of Graziani et al. regression models with site-aware splits is

measured on cohorts from The Cancer Genome Atlas (TCGA), breast cancer (BRCA), colorectal cancer (CRC), liver hepatocellular carcinoma (LIHC), lung adenocarcinoma (LUAD), lung squamous cell cancer (LUSC), gastric cancer (STAD) and endometrial cancer (UCEC) on biomarkers for tumor infiltrating lymphocytes (TIL) regional fraction, proliferation, leukocyte fraction, lymphocyte infiltration signature score (LISS), and stromal fraction. Pearson's r ranges between -1 and 1, where a correlation closer to an extreme indicates a better regression model. The performed statistical test is the two-sided t-test. Significance is reached at $p \leq 0.05$, indicating the correlation is nonzero following the Scipy implementation. Significance is indicated by * $p \leq 0.05$, ** $p \leq 0.01$, *** $p \leq 0.001$, **** $p \leq 0.0001$, or alternatively by ns for $p > 0.05$. Source data are provided as a Source Data file. The yellow color indicates predictions made by the model trained using the method by Graziani et al. . The analysis is performed with independent patient samples, with the number of independent samples indicated within brackets for proliferation, BRCA (687), CRC (266), LIHC (131), LUAD (251), LUSC (280), STAD (201), UCEC (345), for LISS, BRCA (630), CRC (356), LIHC (153), LUAD (201), LUSC (340), STAD (158), UCEC (324), for TIL regional fraction, BRCA (655), CRC (276), LUSC (223), LUAD (269), STAD (149), UCEC (272), for stromal fraction, BRCA (589), CRC (234), LIHC (157), LUAD (294), LUSC (287), STAD (152), UCEC (384), and for leukocyte fraction, BRCA (763), CRC (312), LIHC (159), LUAD (323), LUSC (289), STAD (193), UCEC (317).

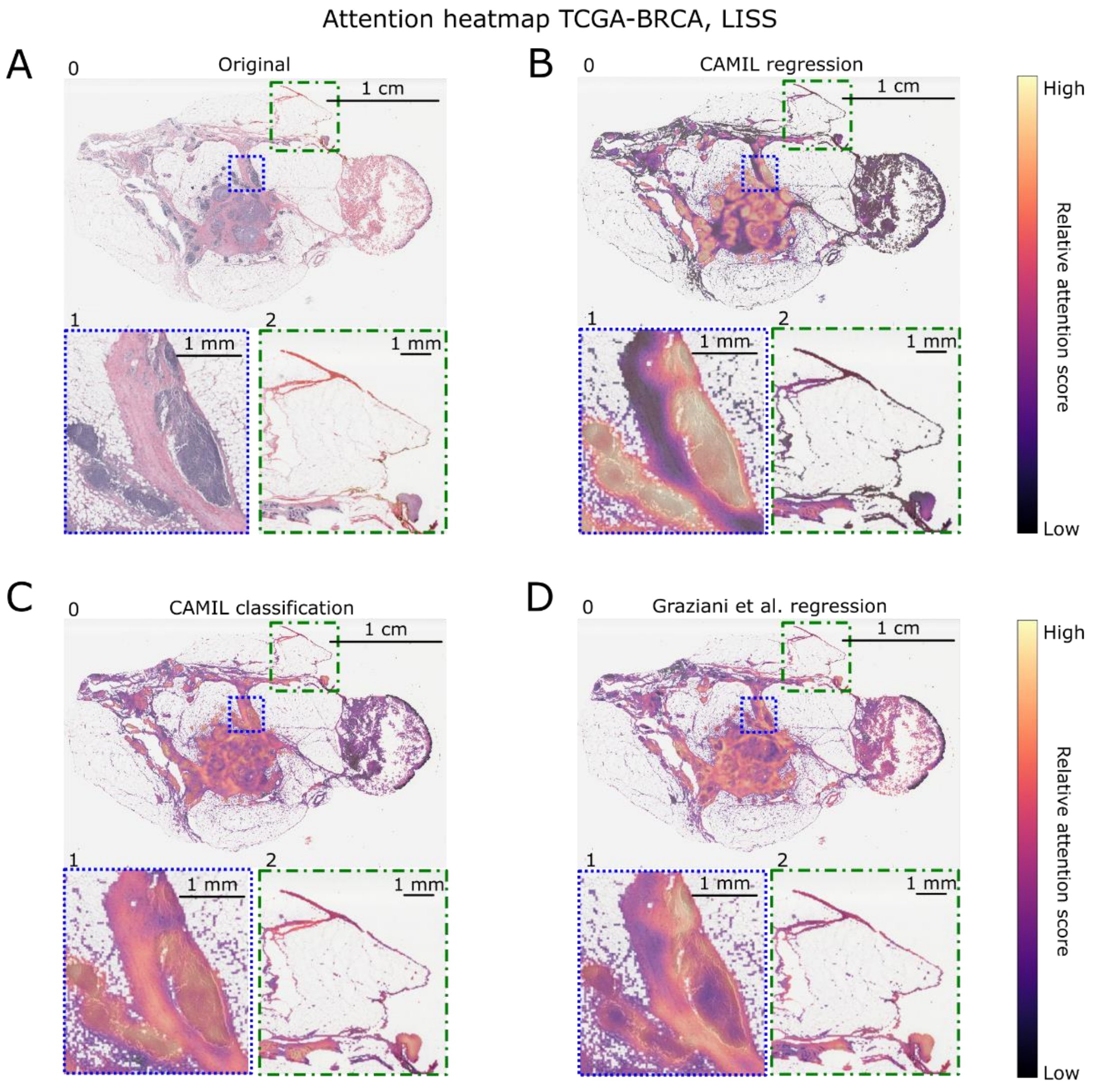

Suppl. Fig. 5: Attention heatmaps of the three modeling approaches for a lymphocyte-based target. Attention heatmap of a slide from the test set of The Cancer Genome Atlas (TCGA) breast cancer (BRCA) cohort. Image 0 shows the entire slide, with an area of interest for diagnostics in image 1. Image 2 shows an area presumably containing non-essential diagnostics information. This is repeated for the original slide in panel A), the attention heatmap using CAMIL regression in panel B), the CAMIL classification model attention heatmap in panel C), and the attention heatmap using the Graziani et al. regression model in panel D) for the lymphocyte infiltrating signature score (LISS). The higher the attention score of an area, the more important it is for the model's decision making.

	TCGA-CRC	DACHS-CRC
Usage	Model training	Overall Survival (OS)
Cohort type	Population	Population
# of patients	632	2448
Median OS (days)	-	3604 [3355 - 3839]
Age (median)	68	69
Age (IQR)	18	14
Sex: Male	322 (50.9%)	1436 (58.7%)
Sex: Female	292 (46.2%)	1012 (41.3%)
Sex: Unknown	18 (2.85%)	0
Stage 1	76 (12%)	485 (19.8%)
Stage 2	166 (26.3%)	801 (32.7%)
Stage 3	140 (22.2%)	822 (33.6%)
Stage 4	63 (10%)	337 (13.8%)
Stage unknown	187 (29.5)	3 (0.1%)
Left-sided CRC	248 (39.2%)	1607 (65.6%)
Right-sided CRC	176 (27.8%)	819 (33.5%)
Unknown side	209 (33%)	22 (0.9%)

Suppl. Table 10: Clinical features of The Cancer Genome Atlas (TCGA) and the Darmkrebs: Chancen der Verhütung durch Screening (DACHS) colorectal cancer (CRC) cohort. The external cohort with CRC patients for the biological process biomarkers came from the DACHS study. The TCGA cohort is utilized for model training, whereas the DACHS cohort is utilized for overall survival (OS) prediction in CRC. The median OS of the DACHS cohort, expressed in days, consists of the 0.95 lower confidence level and the 0.95 higher confidence level. The TCGA-CRC cohort has clinical information for 632 patients, but only whole-slide images for 625 patients are available. The DACHS-CRC cohort has clinical information for 2448 patients, but only 2297 have overlapping whole-slide images and corresponding survival data.

	TCGA-CRC on DACHS-CRC				TCGA-BRCA on DACHS-CRC			
CAMIL model	HR	95%CI Low	95%CI High	p-value	HR	95%CI Low	95%CI High	p-value
Classification – SF	0.24	0.14	0.41	2.28E-07	0.96	0.57	1.59	0.86
Regression – SF	0.30	0.12	0.76	1.05E-02	0.51	0.23	1.12	0.09
Classification – LISS	0.09	0.04	0.18	2.27E-10	0.90	0.61	1.33	0.58
Regression – LISS	0.01	1.84E-03	0.02	2.59E-14	0.14	0.06	0.34	1.34E-05
Classification – LF	0.34	0.23	0.49	2.62E-08	0.48	0.31	0.75	1.06E-03
Regression – LF	0.12	4.00E-02	0.36	1.62E-04	0.10	0.04	0.23	4.39E-08
Classification – Prolif.	2.20	1.30	3.72	3.21E-03	1.16	0.81	1.66	0.41
Regression – Prolif.	0.79	0.11	5.82	0.82	1.82	0.72	4.62	0.20
Classification – TIL RF	0.54	0.37	0.79	1.58E-03	0.63	0.47	0.83	1.08E-03
Regression – TIL RF	0.11	0.04	0.33	1.04E-04	0.13	0.04	0.45	1.33E-03

Suppl. Table 11: Hazard ratios (HR) with 95% confidence interval (CI) and corresponding p-values of univariable Cox proportional-hazards models for the models for biological process biomarkers with continuous scores. The CAMIL regression and CAMIL classification models were trained using site-aware splits on cohorts from The Cancer Genome Atlas colorectal cancer (CRC) and breast cancer (BRCA) on biomarkers for tumor infiltrating lymphocytes regional fraction (TIL RF), proliferation (Prolif.), leukocyte fraction (LF), lymphocyte infiltration signature score (LISS), and stromal fraction (SF). These models were deployed on colorectal patients from the Darmkrebs: Chancen der Verhütung durch Screening (DACHS) study. For the classification models, the predicted dichotomised labels were used, whereas our regression model used the predicted continuous scores for the univariable Cox proportional-hazards models. The p-values and 95%CI are calculated through fitting the Cox's proportional hazard model for each variable independently. An HR confidence interval crossing 1 indicates non-significant prognostication capability. Prognostic capabilities that exhibit a stronger effect can be considered relatively better, as indicated by a HR further away from 1. The sample size to derive statistics is n=2297 independent patient samples for each variable, with n=1345 males (median age 69), n=952 females (median age 70)

CAMIL classification model / Covariates	TCGA-CRC on DACHS-CRC HR	95%CI Low	95%CI High	p-value	TCGA-BRCA on DACHS-CRC HR	95%CI Low	95%CI High	P-value
TIL RF	0.89	0.61	1.30	0.56	0.88	0.66	1.17	0.39
**Prolif.**	**1.95**	**1.16**	**3.29**	**1.18E-02**	**1.44**	**1.00**	**2.06**	**0.05**
SF	0.41	0.24	0.71	1.50E-03	1.03	0.62	1.72	0.91
LF	0.53	0.36	0.78	1.21E-03	0.79	0.50	1.23	0.29
LISS	0.26	0.12	0.56	5.81E-04	1.07	0.72	1.58	0.75
TS 1 vs. 2	1.27	1.05	1.55	1.60E-02	1.27	1.05	1.55	1.54E-02
TS 1 vs. 3	1.59	1.31	1.93	2.03E-06	1.60	1.32	1.93	1.79E-06
TS 1 vs. 4	7.94	6.47	9.74	2.51E-87	7.92	6.46	9.72	9.32E-88
AGE	1.05	1.04	1.06	3.47E-53	1.05	1.04	1.06	6.39E-53
SEX	1.03	0.91	1.16	0.66	1.03	0.91	1.16	0.67

CAMIL regression model / Covariates	TCGA-CRC on DACHS-CRC HR	95%CI Low	95%CI High	p-value	TCGA-BRCA on DACHS-CRC HR	95%CI Low	95%CI High	P-value
TIL RF	0.33	0.11	0.98	4.56E-02	0.37	0.11	1.27	0.11
Prolif.	3.04	0.41	22.46	0.28	1.64	0.66	4.09	0.29
SF	0.45	0.18	1.15	9.42E-02	0.56	0.25	1.24	0.15
LF	0.24	0.08	0.74	1.28E-02	0.26	0.12	0.60	1.35E-03
LISS	0.07	0.02	0.24	3.92E-05	0.32	0.13	0.76	9.77E-03
TS 1 vs. 2	1.27	1.04	1.54	1.67E-02	1.28	1.06	1.56	0.01
TS 1 vs. 3	1.58	1.30	1.91	3.05E-06	1.60	1.32	1.94	1.44E-06
TS 1 vs. 4	7.84	6.39	9.61	3.81E-87	7.93	6.47	9.72	1.14E-88
AGE	1.05	1.04	1.06	6.77E-53	1.05	1.04	1.06	7.03E-53
SEX	1.02	0.90	1.15	0.74	1.02	0.91	1.15	0.73

Suppl. Table 12: Hazard ratios (HR) with 95% confidence interval (CI) and corresponding p-values of multivariable Cox proportional-hazards models with continuous scores for the CAMIL classification and regression models for biological process biomarkers. The CAMIL regression and CAMIL classification models were trained using site-aware splits on cohorts from The Cancer Genome Atlas colorectal cancer (CRC) and breast cancer (BRCA) on biomarkers for tumor infiltrating

lymphocytes regional fraction (TIL RF), proliferation (Prolif.), leukocyte fraction (LF), lymphocyte infiltration signature score (LISS), and stromal fraction (SF). These models were deployed on CRC patients from the Darmkrebs: Chancen der Verhütung durch Screening (DACHS) study. For the classification and regression models the predicted continuous scores were used. The covariates used in the analysis are sex, age, and tumor stage (TS). The p-values and 95%CI are calculated through fitting the Cox's proportional hazard model for each variable independently. An HR confidence interval crossing 1 indicates non-significant prognostication capability. Prognostic capabilities that exhibit a stronger effect can be considered relatively better, as indicated by a HR further away from 1. The sample size to derive statistics is n=2297 independent patient samples for each variable, with n=1345 males (median age 69), n=952 females (median age 70).

	TCGA-CRC on DACHS-CRC				TCGA-BRCA on DACHS-CRC			
Graziani et al. regression model / Covariates	HR	95%CI Low	95%CI High	p-value	HR	95%CI Low	95%CI High	P-value
TIL RF	7.61E-04	2.21E-08	26	0.18	1.36	6.81E-06	2.70E+05	0.96
Prolif.	334	0.03	3.46E+06	0.22	14.97	0.68	329.22	0.09
SF	262	1.46E-05	4.70E+09	0.51	1.85E-03	1.29E-06	2.65	0.09
LF	5.12E+03	0.92	2.85E+07	0.05	3.09E-05	6.75E-09	0.14	0.02
LISS	8.23E-08	2.77E-14	0.24	3.19E-02	0.02	2.24E-05	13.08	0.23
TS 1 vs. 2	1.26	1.03	1.53	0.02	1.27	1.05	1.55	0.01
TS 1 vs. 3	1.56	1.29	1.90	6.20E-06	1.60	1.32	1.94	1.60E-06
TS 1 vs. 4	7.86	6.40	9.64	1.12E-86	8.01	6.54	9.81	1.51E-89
AGE	1.05	1.04	1.06	2.74E-53	1.05	1.04	1.06	5.25E-53
SEX	1.03	0.91	1.16	0.67	1.03	0.91	1.16	0.64

Suppl. Table 13: Hazard ratios (HR) with 95% confidence interval (CI) and corresponding p-values of multivariable Cox proportional-hazards models for the models resulting from Graziani et al. for biological process biomarkers. The Graziani et al. regression models were trained using site-aware splits on cohorts from The Cancer Genome Atlas colorectal cancer (CRC) and breast cancer (BRCA) on biomarkers for tumor infiltrating lymphocytes regional fraction (TIL RF), proliferation (Prolif.), leukocyte fraction (LF), lymphocyte infiltration signature score (LISS), and stromal fraction (SF). These models were deployed on CRC patients from the Darmkrebs: Chancen der Verhütung durch Screening (DACHS) study. The covariates used in the analysis are sex, age, and tumor stage. The p-values and 95%CI are calculated through fitting the Cox's proportional hazard model for each variable independently. An HR confidence interval crossing 1 indicates non-significant prognostication capability. Prognostic capabilities that exhibit a stronger effect can be considered relatively better, as indicated by a HR further away from 1. The sample size to derive statistics is n=2297 independent patient samples for each variable, with n=1345 males (median age 69), n=952 females (median age 70). The HR are inaccurate due to convergence errors while attempting to fit a Cox model, caused by the low variance in the model's predictions due to the Graziani et al. regression model's poor generalizability to an external cohort.

Variable	Sex	Estimate	Std_Error	Statistic	P_Value	Conf_Low	Conf_High
TIL Regional Fraction	Male	0.47	0.26	-2.92	3.55E-03	0.28	0.78
Proliferation	Male	2.11	0.35	2.15	3.19E-02	1.07	4.19
Stromal Fraction	Male	0.22	0.37	-4.12	3.81E-05	0.10	0.45
Leukocyte Fraction	Male	0.24	0.26	-5.43	5.64E-08	0.15	0.40
LISS	Male	0.07	0.52	-5.13	2.84E-07	0.03	0.19

Variable	Sex	Estimate	Std_Error	Statistic	P_Value	Conf_Low	Conf_High
TIL Regional Fraction	Female	0.66	0.29	-1.41	1.60E-01	0.37	1.18
Proliferation	Female	2.44	0.42	2.13	3.33E-02	1.07	5.57
Stromal Fraction	Female	0.26	0.42	-3.20	1.37E-03	0.11	0.59
Leukocyte Fraction	Female	0.50	0.30	-2.32	2.06E-02	0.28	0.90
LISS	Female	0.11	0.59	-3.82	1.35E-04	0.03	0.34

Suppl. Table 14: Univariable Cox proportional-hazards model on predictions from CAMIL classification trained on colorectal cancer samples, disaggregated by sex. The sample size to derive statistics is n=2297 independent patient samples for each variable, with n=1345 males (median age 69), n=952 females (median age 70), for the targets tumor infiltrating lymphocyte (TIL) regional fraction, proliferation, stromal fraction, leukocyte fraction, and the lymphocyte infiltrating signature score (LISS).

Variable	Sex	Estimate	Std_Error	Statistic	P_Value	Conf_Low	Conf_High
TIL Regional Fraction	Male	0.10	0.76	-3.03	2.42E-03	0.02	0.44
Proliferation	Male	2.47	1.34	0.68	4.99E-01	0.18	33.97
Stromal Fraction	Male	0.15	0.63	-3.04	2.34E-03	0.04	0.51
Leukocyte Fraction	Male	0.07	0.76	-3.60	3.17E-04	0.01	0.29
LISS	Male	0.00	0.88	-6.10	1.08E-09	0.00	0.03

Variable	Sex	Estimate	Std_Error	Statistic	P_Value	Conf_Low	Conf_High
TIL Regional Fraction	Female	0.13	0.87	-2.35	1.89E-02	0.02	0.71
Proliferation	Female	0.19	1.59	-1.04	2.98E-01	0.01	4.32
Stromal Fraction	Female	0.72	0.69	-0.48	6.31E-01	0.18	2.79
Leukocyte Fraction	Female	0.25	0.84	-1.63	1.02E-01	0.05	1.32
LISS	Female	0.01	1.00	-4.63	3.60E-06	0.00	0.07

Suppl. Table 15: Univariable Cox proportional-hazards model on predictions from CAMIL regression trained on colorectal cancer samples, disaggregated by sex. The sample size to derive statistics is n=2297 independent patient samples for each variable, with n=1345 males (median age 69), n=952 females (median age 70), for the targets tumor infiltrating lymphocyte (TIL) regional fraction, proliferation, stromal fraction, leukocyte fraction, and the lymphocyte infiltrating signature score (LISS).

HRD	3273							452			
Cohort	TCGA							CPTAC			
Cancer types	BRCA	UCEC	PAAD	CRC	LUAD	GBM	LUSC	UCEC	PAAD	LUSC	LUAD
N slides	1133	566	209	625	544	860	512	883	557	1081	1137
N features	1133	566	209	599	529	860	512	883	557	1081	1125
N target overlap	1005	467	173	496	449	232	451	99	139	108	106
N HRD+	281	68	13	16	158	6	232	3	4	33	14
N HRD-	724	399	160	480	291	226	219	96	135	75	92

Suppl. Table 16: Data availability for the homologous recombination deficiency (HRD) target. Data availability of patients from The Cancer Genome Atlas (TCGA), breast cancer (BRCA), colorectal cancer (CRC), glioblastoma (GBM), lung adenocarcinoma (LUAD), lung squamous cell cancer (LUSC), pancreatic cancer (PAAD) and endometrial cancer (UCEC) for HRD. The external cohorts for HRD were from the Clinical Proteomic Tumor Analysis Consortium (CPTAC) effort, consisting of UCEC, PAAD, LUSC and LUAD.

**LISS**							**3636**	**0**
Cohort	TCGA							DACHS
Cancer types	BRCA	UCEC	STAD	CRC	LUAD	LIHC	LUSC	CRC
N slides	1133	566	209	625	544	860	512	3617
N features	1133	566	209	599	529	860	512	2297
N target overlap	1048	490	334	560	410	331	463	2297
**SF**							**3513**	**0**
Cohort	TCGA							DACHS
Cancer types	BRCA	UCEC	STAD	CRC	LUAD	LIHC	LUSC	CRC
N slides	1133	566	209	625	544	860	512	3617
N features	1133	566	209	599	529	860	512	2297
N target overlap	989	456	360	500	441	320	447	2297
**TIL RF**							**3124**	**0**
Cohort	TCGA							DACHS
Cancer types	BRCA	UCEC	STAD	CRC	LUAD	LIHC	LUSC	CRC
N slides	1133	566	209	625	544	860	512	3617
N features	1133	566	209	599	529	860	512	2297
N target overlap	943	447	335	555	459	0	385	2297
**LF**							**3719**	**0**
Cohort	TCGA							DACHS
Cancer types	BRCA	UCEC	STAD	CRC	LUAD	LIHC	LUSC	CRC
N slides	1133	566	209	625	544	860	512	3617
N features	1133	566	209	599	529	860	512	2297
N target overlap	1035	493	373	561	459	333	465	2297
**Prolif.**							**3636**	**0**
Cohort	TCGA							DACHS
Cancer types	BRCA	UCEC	STAD	CRC	LUAD	LIHC	LUSC	CRC
N slides	1133	566	209	625	544	860	512	3617
N features	1133	566	209	599	529	860	512	2297
N target overlap	1048	490	334	560	410	331	463	2297

Suppl. Table 17: Data availability for the biomarkers related to biological processes. Data availability of patients from The Cancer Genome Atlas (TCGA), breast cancer (BRCA), colorectal cancer (CRC), liver hepatocellular carcinoma (LIHC), lung adenocarcinoma (LUAD), lung squamous cell cancer (LUSC), gastric cancer (STAD) and endometrial cancer (UCEC) for biological process biomarkers: tumor infiltrating lymphocytes regional fraction (TIL RF), proliferation (Prolif.), leukocyte fraction (LF), lymphocyte infiltration signature score (LISS), and stromal fraction (SF). The external

cohort with CRC patients for the biological process biomarkers came from the Darmkrebs: Chancen der Verhütung durch Screening (DACHS) study.

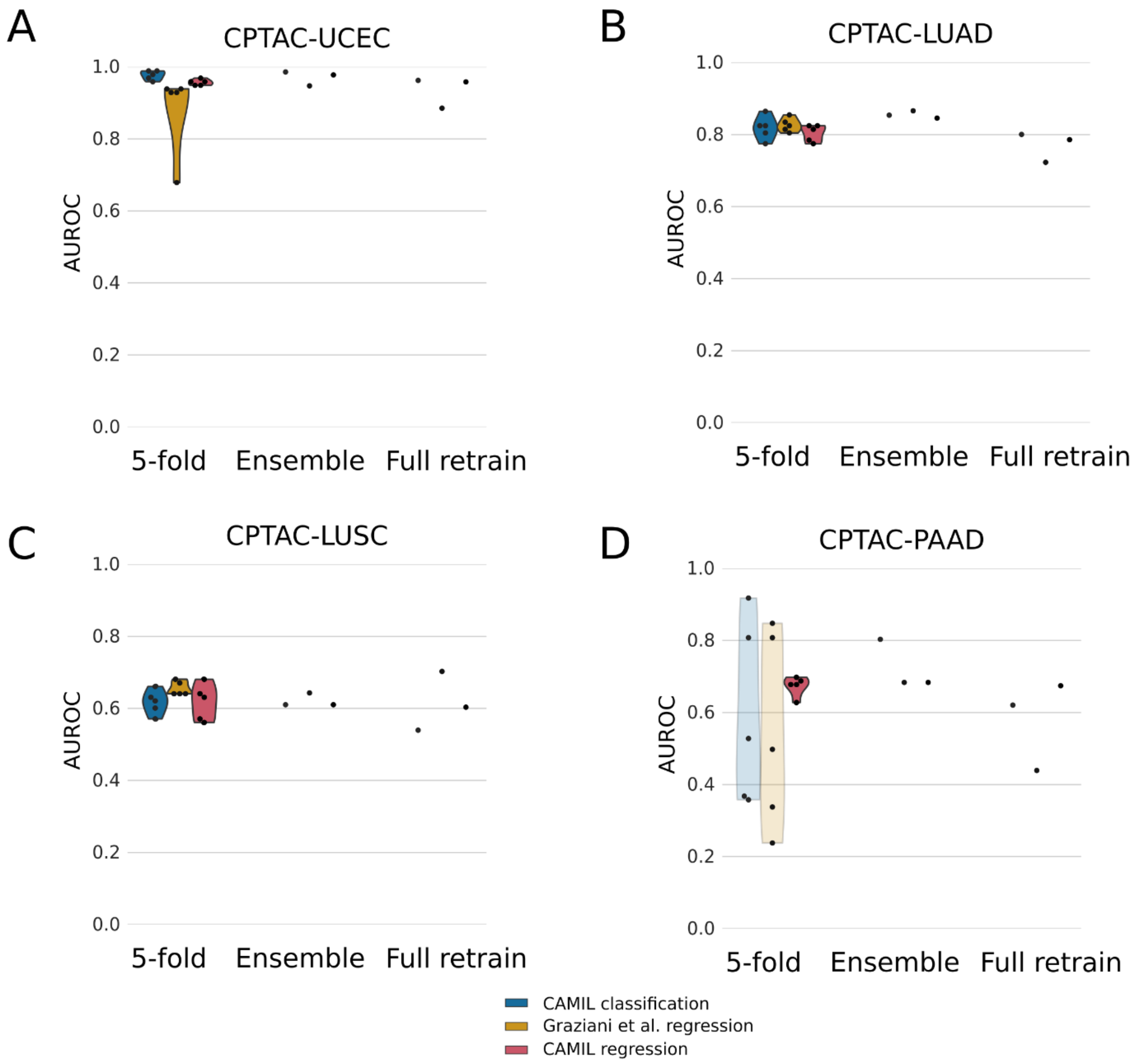

Suppl. Fig. 6: Performance overview of external deployment of homologous recombination deficiency (HRD) models using the 5-folds, median ensemble, and full retraining method. The three modeling approaches were trained on The Cancer Genome Atlas (TCGA) for lung adenocarcinoma (LUAD), lung squamous cell cancer (LUSC), pancreatic adenocarcinoma (PAAD) and endometrial cancer (UCEC). The models were then deployed on the Clinical Proteomic Tumor Analysis Consortium (CPTAC) cohorts, showing the results of UCEC in panel A, LUAD in panel B, LUSC in panel C, and PAAD in panel D. The 5-folds were directly deployed from the respective 5-fold models from the TCGA training cohort, generating 5 AUROCs per modeling approach for each cancer type. The ensemble was performed by taking the median prediction score of the 5 models, generating 1 AUROC per modeling approach for each cancer type. The full retraining was performed by retraining a new model on 100% of the TCGA training cohorts and deploying it on the CPTAC cohorts, generating 1 AUROC per modeling approach for each cancer type. Source data are provided as a Source Data file.
