# Supplementary material for: Regression-based Deep-Learning predicts molecular biomarkers from pathology slides
Source: Nat Commun. 2024 Feb 10;15:1253. doi: 10.1038/s41467-024-45589-1 (PMC10858881; doi:10.1038/s41467-024-45589-1)
Supplement: Supplementary file 3 — Reporting Summary [file 41467_2024_45589_MOESM3_ESM.pdf]

nature portfolio

Corresponding author(s): Jakob Nikolas Kather

Last updated by author(s): Jan 26, 2024

Reporting Summary

Nature Portfolio wishes to improve the reproducibility of the work that we publish. This form provides structure for consistency and transparency in reporting. For further information on Nature Portfolio policies, see our Editorial Policies and the Editorial Policy Checklist.

Statistics

For all statistical analyses, confirm that the following items are present in the figure legend, table legend, main text, or Methods section.

n/a	Confirmed	
☐	☒	The exact sample size (*n*) for each experimental group/condition, given as a discrete number and unit of measurement
☐	☒	A statement on whether measurements were taken from distinct samples or whether the same sample was measured repeatedly
☐	☒	The statistical test(s) used AND whether they are one- or two-sided<br>*Only common tests should be described solely by name; describe more complex techniques in the Methods section.*
☐	☒	A description of all covariates tested
☐	☒	A description of any assumptions or corrections, such as tests of normality and adjustment for multiple comparisons
☐	☒	A full description of the statistical parameters including central tendency (e.g. means) or other basic estimates (e.g. regression coefficient) AND variation (e.g. standard deviation) or associated estimates of uncertainty (e.g. confidence intervals)
☐	☒	For null hypothesis testing, the test statistic (e.g. *F*, *t*, *r*) with confidence intervals, effect sizes, degrees of freedom and *P* value noted<br>*Give P values as exact values whenever suitable.*
☒	☐	For Bayesian analysis, information on the choice of priors and Markov chain Monte Carlo settings
☐	☒	For hierarchical and complex designs, identification of the appropriate level for tests and full reporting of outcomes
☐	☒	Estimates of effect sizes (e.g. Cohen's *d*, Pearson's *r*), indicating how they were calculated

Our web collection on statistics for biologists contains articles on many of the points above.

Software and code

Policy information about availability of computer code

Data collection	No software was used for the collection of data.
Data analysis	All source codes are available under an open-source license on GitHub. The pre-processing pipeline is found at https://github.com/KatherLab/end2end-WSI-preprocessing/releases/ tag/v1.0.0-preprocessing, the classification pipeline is found at https://github.com/KatherLab/marugoto/releases/tag/v1.0.0-classification, the regression pipeline is found at https://github.com/KatherLab/marugoto/releases/tag/v1.0.0-regression, and the classification and regression attention heatmaps are found at https://github.com/KatherLab/highres-WSI-heatmaps/releases/tag/v1.0.0-heatmaps.

For manuscripts utilizing custom algorithms or software that are central to the research but not yet described in published literature, software must be made available to editors and reviewers. We strongly encourage code deposition in a community repository (e.g. GitHub). See the Nature Portfolio guidelines for submitting code & software for further information.

Data

Policy information about availability of data

All manuscripts must include a data availability statement. This statement should provide the following information, where applicable:

- Accession codes, unique identifiers, or web links for publicly available datasets
- A description of any restrictions on data availability
- For clinical datasets or third party data, please ensure that the statement adheres to our policy

The slides for TCGA are available at https://portal.gdc.cancer.gov/. The slides for CPTAC are available at https://proteomics.cancer.gov/data-portal. The molecular data for TCGA and CPTAC are available at https://www.cbioportal.org/ and additional biomarker data is available from Thorsson et al.36 The slides and biomarker data for DACHS were generated for prior studies62–64 with restricted access. Biomarker data for DACHS are available by requesting Authorized Access to the phs001078 study (https://www.ncbi.nlm.nih.gov/projects/gap/cgi-bin/study.cgi?study_id=phs001113.v1.p1). Applications for access to DACHS biomarker data are reserved for Senior Investigators and NIH Investigators as defined in https://dbgap.ncbi.nlm.nih.gov/aa/wga.cgi, and upon successful application grants access to the data for 1 year with the option to renew access. The slides for DACHS can only be requested directly through the DACHS principal investigators. The contact details are listed at http://dachs.dkfz.org/dachs/kontakt.html. Written informed consent was obtained by participants in DACHS. Participants received no compensation for participation. The data generated in this study for the creation of the figures are provided in the Source Data file.

Research involving human participants, their data, or biological material

Policy information about studies with human participants or human data. See also policy information about sex, gender (identity/presentation), and sexual orientation and race, ethnicity and racism.

Reporting on sex and gender	We report sex-specific information in supplementary table 10, and disaggregated results by sex in supplementary table 14 and 15.
Reporting on race, ethnicity, or other socially relevant groupings	The country of origin for cohort TCGA is the US, for cohort CPTAC is the US, for cohort DACHS is Germany. No information about race was available.
Population characteristics	For the survival analysis performed on the DACHS cohort, the cohort has 2448 patients with a median overall survival (with 0.95 lower and upper confidence levels) of 3604 [3355-3839] days. The median age and interquartile range of the participants are 69 and 14, respectively. Of the 2448 participants, 1436 patients (58.7%) are male and 1012 patients (41.3%) are female, 485 are stage I (19.8%), 801 are stage II (32.7%), 822 are stage III (33.6%), 337 are stage IV (13.8%) and 3 are unknown (0.1%). Additional information is found in supplementary table 10.
Recruitment	All studies contain samples which were collected with the intent of performing translational research.
Ethics oversight	We examined anonymized patient samples from several academic institutions in this investigation. The collection and analysis of samples in the DACHS cohort was approved by the local ethics board. CPTAC and TCGA did not require formal ethics approval for a retrospective study of anonymised samples. The overall analysis was approved by the Ethics commission of the Medical Faculty of the Technical University Dresden (BO-EK-444102022).

Note that full information on the approval of the study protocol must also be provided in the manuscript.

Field-specific reporting

Please select the one below that is the best fit for your research. If you are not sure, read the appropriate sections before making your selection.

☒ Life sciences ☐ Behavioural & social sciences ☐ Ecological, evolutionary & environmental sciences

For a reference copy of the document with all sections, see nature.com/documents/nr-reporting-summary-flat.pdf

Life sciences study design

All studies must disclose on these points even when the disclosure is negative.

Sample size	No explicit sample-size calculation was performed. Recent studies in computational pathology by Foersch et al. (Nat Med, 2023) and Wagner et al. (Cancer Cell, 2023) showed successful biomarker predictions with cohorts having several hundred patients, with larger cohorts yielding better results. Consequently, we collected datasets which satisfied this volume range of patients, having several hundreds, or thousands of patients available for retrospective analysis. Moreover, a recent preprint by El Nahhas et al. provided rough guidelines for an estimation of sample sizes for computational pathology (https://arxiv.org/abs/2312.10944v1).
Data exclusions	In all experiments, data samples were excluded when the microns-per-pixel information was not available in the metadata of the whole-slide image, or if the biomarker to be predicted was not available for the sample. More details are found in the methods and supplementary table 16 and 17.
Replication	Random seeds set in the code, patient splits for training and testing models were saved, and the code version was managed through GitHub for reproducibility.

Randomization	Samples were stochastically allocated into different groups through site-aware data stratification, where the model was trained, validated and tested on 1) different patients, and 2) different hospitals. More details in the methods and accompanying code.
Blinding	The performed study is retrospective. The data is split into training and validation datasets. The TCGA is used as training cohort. For validation, CPTAC and DACHS are analyzed in a blinded manner so that no data leakage occurs between the training and validation cohorts. Therefore, the trained models only contain insights about the TCGA cohort, without learning from the blinded CPTAC and DACHS cohorts.

Reporting for specific materials, systems and methods

We require information from authors about some types of materials, experimental systems and methods used in many studies. Here, indicate whether each material, system or method listed is relevant to your study. If you are not sure if a list item applies to your research, read the appropriate section before selecting a response.

Materials & experimental systems

n/a	Involved in the study
☒	☐ Antibodies
☒	☐ Eukaryotic cell lines
☒	☐ Palaeontology and archaeology
☒	☐ Animals and other organisms
☒	☐ Clinical data
☒	☐ Dual use research of concern
☒	☐ Plants

Methods

n/a	Involved in the study
☒	☐ ChIP-seq
☒	☐ Flow cytometry
☒	☐ MRI-based neuroimaging

Plants

Seed stocks	*Report on the source of all seed stocks or other plant material used. If applicable, state the seed stock centre and catalogue number. If plant specimens were collected from the field, describe the collection location, date and sampling procedures.*
Novel plant genotypes	*Describe the methods by which all novel plant genotypes were produced. This includes those generated by transgenic approaches, gene editing, chemical/radiation-based mutagenesis and hybridization. For transgenic lines, describe the transformation method, the number of independent lines analyzed and the generation upon which experiments were performed. For gene-edited lines, describe the editor used, the endogenous sequence targeted for editing, the targeting guide RNA sequence (if applicable) and how the editor was applied.*
Authentication	*Describe any authentication procedures for each seed stock used or novel genotype generated. Describe any experiments used to assess the effect of a mutation and, where applicable, how potential secondary effects (e.g. second site T-DNA insertions, mosiacism, off-target gene editing) were examined.*
